# The evolution of barriers to exploitation: Sometimes the Red Queen can take a break

**DOI:** 10.1111/eva.13280

**Published:** 2021-08-04

**Authors:** Jonathan R. Goodman, Paul W. Ewald

**Affiliations:** ^1^ Leverhulme Centre for Human Evolutionary Studies University of Cambridge Cambridge UK; ^2^ Department of Biology University of Louisville Louisville Kentucky USA

**Keywords:** barrier theory, coevolution, evolutionary ecology, evolutionary medicine, exploitation, Red Queen

## Abstract

We propose a general barrier theory as an evolutionary framework for understanding coevolutionary effects of conflicts of interest in natural and human systems. It is generalized from the barrier theory of cancer, which describes how cancer develops through the evasion of mechanisms, that block unregulated cellular reproduction and survival. Barriers are naturally evolved or artificially implemented mechanisms for blocking exploitation; restraints are mechanisms that impede but do not block exploitation. When conflicts of interest arise, selection will favor exploiters that are capable of overcoming barriers and restraints. When barriers are in place, they halt, at least temporarily, coevolutionary arms races (the Red Queen can stop running). Barriers occur in a broad spectrum of interactions characterized by conflicts of interest: barriers to cellular survival (apoptosis) and reproduction (cell cycle arrest) may block a virus from replicating its genome through reproduction of its host cell. Vaccines may completely protect against targeted pathogens. A plant may escape herbivory by evolving defensive chemicals that block herbivory. Obligate mutualisms may evolve when barriers to horizontal transmission favor symbionts that increasingly lose mechanisms that contribute to horizontal transmission. Here, we show how the barrier theory applies across a spectrum of natural and social systems.

## 1 INTRODUCTION

The barrier theory of oncogenesis (Ewald and Swain Ewald, 2013) offers an evolutionary framework based on the conflicts of interest between a cell acting in its own short‐term reproductive interest and the evolutionary fitness of the multicellular organism in which the cell resides. It proposes that the diverse and complex interactions of oncogenesis within a person and among cancers can be usefully organized by identifying the few cellular processes that block a cell's pathway to cancer (e.g., cell cycle arrest and cell suicide). These barriers are distinguished from the many restraints that may slow but do not block oncogenesis (e.g., slow division of dividing cells, restricted blood flow). These distinctions allow the essential causes of cancer, which circumvent barriers, to be distinguished from the many exacerbating causes, which compromise restraints. Oncogenic viruses evolve to circumvent barriers to cancer because barriers to cancer are also barriers to the survival and reproduction of viral genomes within the cell.

The distinction between barriers and restraints can be applied more broadly to any situation in which there are conflicts of interest and possibilities for exploitation and for defenses against exploitation. The extent to which barriers can be maintained over time specifies conditions in which coevolutionary arms races can be halted. This implies either the creation of a new evolutionarily stable strategy or of the loss of an arms race; the coevolutionary relationship will not restart unless a strategy is developed for evading the barrier.

Van Valen (1973) focused on how this ongoing antagonistic coevolutionary process could lead to regularity in rates of species extinctions. Building on Hamilton’s (1980) idea that sexual reproduction could allow hosts to stay ahead of parasites in their evolutionary arms races, Bell (1982) expanded the Red Queen concept to fluctuations in the success of genetic variants as an explanation for the evolutionary maintenance of sexual reproduction. These and subsequent applications of the Red Queen hypothesis to the evolution of sex have emphasized genotype oscillations over time (Kouyos, 2007; Lively, 2010), but cyclic dynamics are not necessary for the continuation of the coevolutionary process; genetic diversity per se can be sufficient (Ashby, 2020; Lively, 2010), and the process can involve directional selection (Brockhurst et al., 2014).

We consider the Red Queen broadly to include any coevolutionary process through which the interactants are persistently changing in response to each other. These processes could involve cycles of allele and genotype frequencies or ongoing coevolutionary changes that are not cyclic. Noncyclic changes could occur when the defenses are beneficial because offspring differ from the parents or siblings that can transmit exploiters (Aubier et al., 2020; Greenspoon and Mideo, 2017), or as a result of epidemiological influences (MacPherson & Otto, 2018).

**FIGURE 1:**
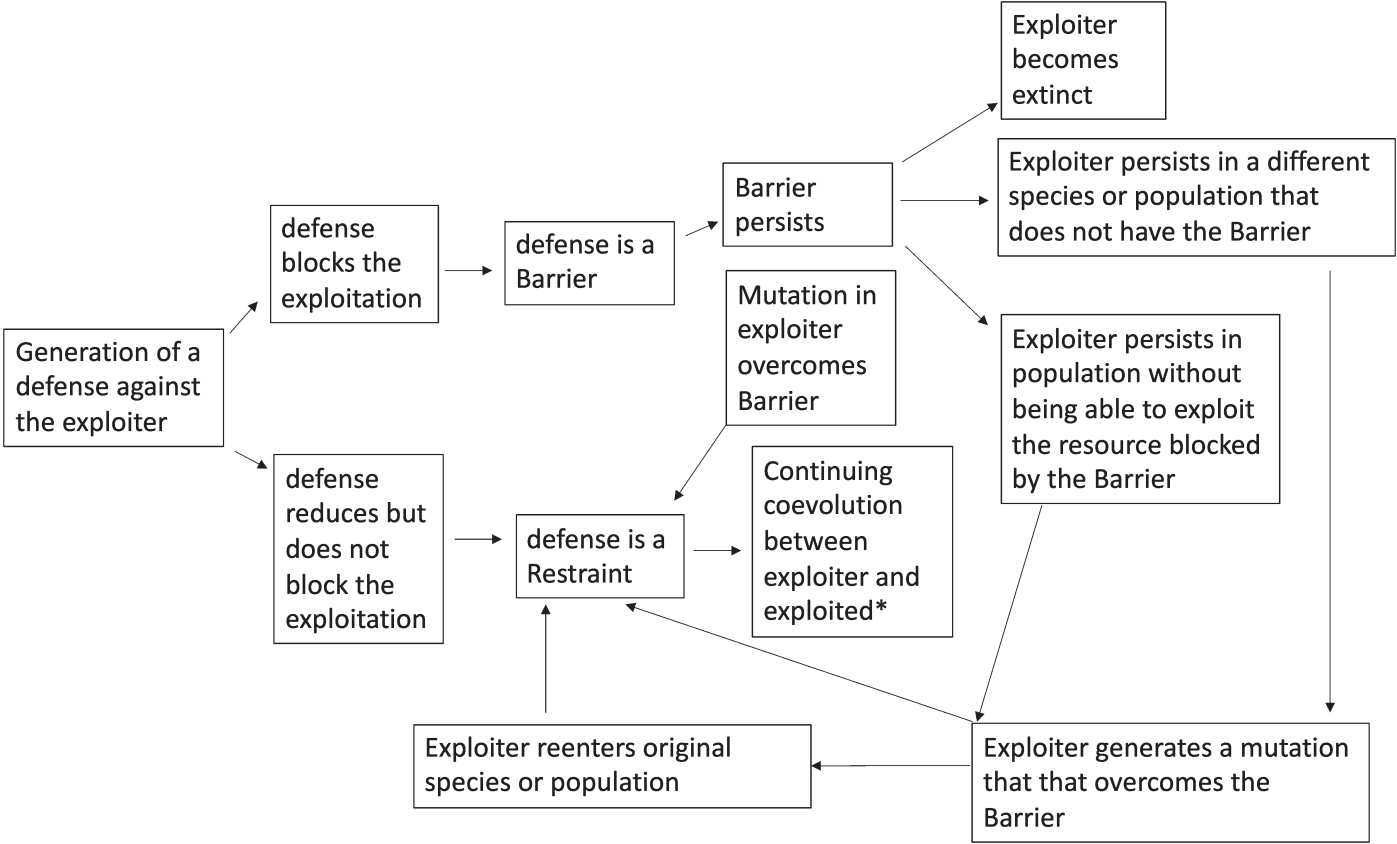
The general barrier theory of coevolution. The compromising of barriers here is assumed to be due to mutations but could be generated by other means, for example, in the process of cultural evolution. The asterisk indicates where the Red Queen (broadly defined) continues to run. See text for further explanation

The barrier theory helps to structure the overall applicability of the Red Queen hypothesis because ongoing cyclic dynamics result when defenses against exploitation are restraints—the defenses suppress rather than block the antagonist. If the coevolutionary changes are not cyclic, the defenses could be restraints if the genetic variation to overcome the defense is present in the exploitative population, or barriers if the exploiter population does not have the genetic information needed to counter the defense. In the latter case, the exploiter population could become extinct or switch to another population of the same or different species. Or the exploiter could persist in the original population but be unable to exploit the host in a way that is blocked by the barrier (e.g., a virus unable to stimulate host cell proliferation because of a cell cycle arrest barrier may be able to replicate is genome through by virion production but not through cellular replication). If the ability to break through the barrier is subsequently generated (e.g., through a new mutation), then the newly acquired ability transforms the barrier into a restraint and coevolutionary process can resume. These processes are diagrammed in Figure 1. The literature on the Red Queen hypothesis focuses on restraints rather than barriers. Consideration of barriers, however, is important because it frames the conditions under which the Red Queen processes will or will not be occurring (as noted by the asterisk in Figure 1). Restraints keep the Red Queen running, whereas barriers allow her to stop, at least temporarily.

We suggest, using illustrations from a spectrum of exploitative relationships, that both barriers and restraints are widespread in natural and cultural systems, though in the latter, the information will be social or acquired, rather than genetic (Borgerhoff Mulder et al., 2006; Boyd & Richerson, 1985). In each context, barriers evolve, in the genetic or cultural sense, to prevent exploitation. Just as the barrier theory of oncogenesis implies prolonged, complex coevolutionary relationships between hosts and oncogenic viruses, selection is similarly likely to favor strategies for thwarting exploitation in and among free‐living organisms, and counterstrategies for overcoming such defenses. As is the case with the barrier theory of oncogenesis, a barrier to exploitation among free‐living organisms stops the coevolutionary process (Figure 1), and breaking of the barrier is an essential cause of the restarting of the coevolutionary process. As is the case with oncogenesis, countermeasures against restraints are exacerbating causes that perpetuate the coevolutionary process; they exacerbate the exploitation and hence increase the selective pressure on the exploited individual to evolve additional protection.

Several claims follow from these arguments, if valid: First, barrier theory, as applied to different ecological and cultural environments, will allow for predictions of how exploitation is likely to arise between competitive individuals or populations (for discussion of parallels between oncologic systems and other ecological and social systems, see Aktipis et al., 2015; Aktipis, 2020). Second, deception and mimicry are likely to be common forms of breaking or overcoming barriers. Third, in human systems, as cultures become larger and more stratified, both the strategies for exploitation and the barriers for preventing them will become more complex. We discuss the relevance of this perspective in parasite/host systems, nonhuman animal signaling systems, and human social systems.

## 2 PARASITISM

Oncogenic viruses may have evolved to evade barriers because this ability increases the chances that the viruses can multiply persistently through proliferation of their host cells (Ewald and Swain Ewald, 2013). The countermeasures that tumor viruses evolved against barriers to oncogenesis transformed the barriers into restraints on both oncogenesis and viral persistence. More generally, when parasites evolve countermeasures that compromise but do not eliminate barriers to exploitation, the barriers become restraints for those parasites. This situation accords with the need for the Red Queen to begin moving to improve the effectiveness of a restraint on exploitation and, if possible, transform a restraint back into a barrier against the coevolving parasite.

Vaccination can be a barrier when the genetic variation in the target pathogen population is insufficient to generate vaccine escape (e.g., smallpox vaccination). When sufficient variation for vaccine escape is present, the vaccination is a restraint (e.g., influenza vaccination). In some cases, vaccination could be a restraint that fundamentally changes the coevolutionary landscape; for example, diphtheria vaccination is barrier against phage‐encoded toxin but a restraint for the *C*. *diphtheriae* that host the phage, resulting in predomination by nontoxigenic *Corynebacterium* *diphtheriae* that are relatively benign for humans (Ewald, 1994).

Genetic defenses against parasites generally are restraints. The allele that causes sickle cell anemia, for example, reduces the mortality from infections with the malaria parasite, *Plasmodium falciparum*, among people who are heterozygous for the allele by suppressing the reproduction of *P*. *falciparum*, but the allele does not prevent disease or infection (Archer et al., 2018). Genetic defenses against parasites can sometimes be barriers, however. *Plasmodium vivax* uses the Duffy antigen receptor to enter cells (Salinas and Tolia, 2016). Mutations in this receptor therefore block the entry of *Plasmodium vivax* (Salinas and Tolia, 2016). The effectiveness of this barrier may explain the absence of *P*. *vivax* from areas in West Africa where the Duffy antigen receptor is highly prevalent. This negative correlation between the prevalence of *P*. *vivax* and the Duffy antigen mutation may have arisen because *P*. *vivax* was once common in Western Africa, but virtually disappeared when the prevalence of the receptor mutation increased to near fixation. Some *P*. *vivax* strains, however, have evolved a workaround, so that individuals with the receptor mutation can be infected (Golassa et al., 2020), abrogating the barrier and restarting a local evolutionary arms race between humans and *P*. *vivax* in individuals who carry the Duffy antigen receptor mutation.

Studies of immunological defenses against parasitism generally focus on the action of immunological defenses against specific parasitic organisms. Evolutionarily these interactions are often viewed in the context of arms races, with immunological adaptations such as leukocyte clonal diversity, antigen presentation, and somatic hypermutation being interpreted as adaptations that allow facultative responses to rapidly evolving adversaries. The flip side of these immunological responses is that they block a great spectrum of micro‐organisms that would otherwise rapidly multiply and damage the host. Opportunistic infections in immunologically compromised hosts, for example, reveal the importance of immunological barriers to potentially damaging micro‐organisms. The distinction between barriers and restraints is therefore central to understanding the function of the immune system because viewing immunological defenses solely in the context of the coexisting parasites that are inhibited but not blocked by the defenses would understate the selection pressure favoring the defense and the benefit of the defense to the host.

Knowledge about the Duffy antigen receptor illustrates this point. The functional Duffy antigen receptor is involved in immunological signaling and the coagulation response to bacterial lipopolysaccharide (Mayr et al., 2009). Evidence indicates that the absence of the Duffy antigen on red blood cells may dampen the coagulation response to bacteria and inhibit tumor development and metastasis (Pruenster et al., 2009). If evolutionary considerations of the mutations were focused only on their altering responses to coagulation and cancer, the most dramatic effect would have been overlooked: their role in creating a barrier to infection by *P. vivax*. Although this effect was apparent to malaria researchers considering genetic defenses across geographic regions, the effects of barriers may often be inconspicuous because the parasites that are blocked by the barrier are only apparent by their absence. A researcher studying the Duffy antigen mutation only in West Africa might fail to notice its effect on *P*. *vivax* because this parasite, being blocked by the mutation, is not present in the study population. The effects of a barrier in other situations may go unnoticed if researchers do not consider why potential exploiters are not exploiting in a given system.

## 3 PREDATION AND HERBIVORY

A major category of interactions in nature involves interactions between free‐living consumers and consumed species. This category encompasses interactions between herbivores and plants, and predators and their prey. Defenses against consumers often involve secondary chemicals or physical structures. A particular defense may be a stable barrier against most consumers but a restraint for small subset of coevolving consumers. Defensive chemicals of pine trees, for example, are barriers for most herbivorous insects. *Dendroctenus* pine beetles, however, have evolved adaptations that not only counter the defenses but also to use the chemicals to home in on vulnerable trees and as a basis for their pheromonal communication; these adaptations allow the beetles to engage in concerted beetle attacks on the trees (Franceschi et al., 2005; Tittiger & Blomquist, 2017). The pine beetles have apparently gained the upper hand in the coevolutionary arms race, so that the chemical defenses are no longer a deterrent even though they certainly must continue to be an effective barrier against a large number of herbivorous insects.

The current deforestation of conifers suggests how a change in the environment, in this case due to global warming, can change the outcome of coevolutionary interactions when barriers have been circumvented. Global warming has apparently shifted the associations from Red Queen coexistence to decimation of the conifer forests by reducing resistance of trees and lengthening the season suitable for beetle reproduction (Bentz et al., 2010; Huang et al., 2020). This decimation has not been attributed to opportunistic herbivores, for which the chemical defenses still function as barriers.

As is the case with the Duffy antigen mutations, the importance of the secondary compounds need to be analyzed not only in the context of the coevolutionary arms race between the pines and the beetles, but also in the context of the herbivores that do not exploit the pine trees because they are entirely deterred by the secondary compounds. This deterrence may be the main explanation for why the investment in secondary compounds is favored by natural selection. Considerations of plant–herbivore arms races by focusing on herbivores that restrained but not blocked may therefore provide an inadequate understanding of how natural selection favors the evolution of secondary compounds.

## 4 MUTUALISMS

The barrier theory is also applicable to the evolution of mutualisms. One application involves modes of transmission of symbionts, defined broadly here to include any organism living in intimate association with a host organism, inclusive of parasites, mutualists, and commensals. If a parasite loses its ability to be horizontally transmitted and is thus transmitted only vertically from parent to offspring, its genetic interest coincides with that of its host. When horizontal transmission is no longer possible, any of the additional adaptations for horizontal transmission become a liability and thus will be selected against and attributes that benefit the hosts will be selected for. The genetic concordance of interests between vertically transmitted symbiont and host should favor further adaptations of the symbiont that benefit the host and vice versa, leading to ever stronger obligate mutualisms, such as the evolution of organelles. Of course, when this process begins, horizontal transmission could be reinstated by compensatory mutations, but the variety of mutations that can result in the disintegration of the potential for horizontal transmission will be greater than the number that can reinstate it. Consequently, we can expect that the evolutionary trajectory after the loss of horizontal transmission will tend to be toward more robust barriers to horizontal transmission and increasingly mutualistic interactions so long as there are benefits that the symbiont can provide to the host.

If the dividing line between parasitism and mutualism (i.e., commensalism; see Swain Ewald and Ewald, 2020) is not crossed, a vertically transmitted parasitic lineage will become extinct because the costs inflicted by the parasite on the host will lower host fitness below that of unparasitized competitors. If the process begins with a marginal mutualism instead of parasitism, loss of horizontal transmission will favor mutualisms that generate ever greater net benefits to the host up to the maximum net benefit, without the time constraint imposed by extinction of a parasitic lineage that is entirely vertically transmitted. Regardless of the starting point, an insurmountable barrier to horizontal transmission of mutualists allows the Red Queen to stop. Accordingly, chloroplasts and mitochondria have never, to our knowledge, evolved to be parasitic. Overall, barriers to horizontal transmission are barriers to host exploitation that increase in strength as a result of ever greater specialization for vertical transmission.

## 5 NONHUMAN SOCIAL BEHAVIOR

Vertical and horizontal information transmission apply similarly in the social systems of both nonvertebrates, bird species, and primates (Boyd & Richerson, 1985; Whiten, 2021). Accordingly, barriers may shift from purely biological to cultural, depending on the population in question. Yet because of the way information is transferred in social systems—notably by imitation and learning (Boyd & Richerson, 1985)—the modes of both barrier formation and exploitation are different in kind from those of biological systems. In social systems, where signals and cues are used by individuals to predict the behaviors of others (Grafen, 1990; Dawkins and Krebs, 1978; Zahavi, 1975) and may evolve through cultural evolution as well as biological evolution (Owren et al., 2010), honest and dishonest signals will provide the information essential for predicting and blocking exploitation. As in purely biological ecologies, the risk of detection and the associated costs of being detected form the costs to mimics.

In nonhuman animals, mimicry of honest signals can be used to exploit social systems, creating a need for social policing (Maynard‐Smith and Harper, 2003), which may act as a barrier or restraint on signal mimicry. The difficulty of replicating the signal in question is, furthermore, a restraint, where at the far extreme—where a signal is not fakeable (referred to variously as a “performative signal” or an “index”; Maynard‐Smith and Harper, 2003). Selection will favor both mimics capable of imitating hard to fake signals, as well as individuals capable of detecting fakery.

For example, in an empirical iteration of the Sir Philip Sydney Game, young, related birds in a single nest use varying calling strategies in an attempt to win a feeding parent's attention (Hutteger and Zollman, 2010; Jamie et al., 2020; Maynard‐Smith, 1994). Chicks that express a greater need for food are more likely to be fed by the adults, though a particular calling strategy does not necessarily indicate a greater need for food.

Exploitation is, in this case, when a chick signals a need for food when there is no such need, thereby mimicking those with a real need; countermeasures against such exploitation are employed by parents that preferentially respond to honest signalers. Insofar as a parent can always recognize and avoid a cheating chick, this ability to perceive correctly is a barrier to cheating; honest signals of need would, in this case, be an index. If, however, recognition is only sometimes successful, the response would be a restraint. Where cheating is observed, we would expect that chicks that need food would signal more desperately, and, further, that parents would have a high sensitivity for honest and dishonest signals—but in the absence of barriers, a restraint may lead only to further exploitative strategies developed by cheaters.

Red Queen‐like coevolutionary relationships in nonhuman animals determine how barriers to exploitation and within‐group subversion evolve in social groups. Some nonhuman animal populations are, for example, observed punishing dishonest signalers. Bachmann et al. (2017) show that even the potential of dishonest signaling can be costly; in the cichlid fish *Neolamprologus brichardi*, unreliable signalers—who misrepresent messages using facial color patterns—tend to be punished, leading to “social policing” that helps to support a cooperative breeding system; policing, in this case, is an example of a socially created restraint, as potential punishment deters but probably cannot entirely prevent exploitation. Tibbetts and Dale (2004) come to similar conclusions among paper wasps on the basis of experimental manipulation of the signal.

Territorial defense of breeding grounds by males may serve as a barrier to mating by competing males when the competitors have not evolved an alternative mating strategy. An alternative mating strategy represents circumvention of the barrier. In centrarchid sunfish *Lepomis macrochirus*, for example, small males avoid male expulsion and thus gain mating access to females on the territories of males by mimicking females (Dominey, 1980; Phillipp & Gross, 1994). In other cases, the extent to which the barrier of territory defense has been evaded is less clear. In red‐winged blackbirds, (*Agelaius phoeniceus*), female mimicry has not been clearly established. Subadult males, however, have coloration that resembles females, though it is often intermediate between the most brightly colored females and the far more brightly colored adult males. Presentation of a mounted specimen of one such intermediately colored subadult male elicited an intense courtship display from the territory owner (Rohwer, 1978), indicating that the territorial defense response has been countered by subadult males. Whether the subadult males might gain immediate mating benefits (as is the case with *L*. *macrochirus*) or the more long‐term advantages (such as a toe‐hole in territory establishment) from the apparent female mimicry is unclear.

Genetic testing to assess whether extra‐pair copulations contribute genetically to offspring might help clarify whether territorial barriers have been evaded. When extra‐pair copulations do not occur, territorial defense can be considered a barrier and selection for coevolutionary changes in territoriality would be lessened, though other factors may contribute to the absence of extra‐pair copulations. The pervasiveness of extra‐pair copulations among species with breeding territories (Brouwer & Griffith, 2019) suggests that territorial barriers are often circumvented and that intraspecific coevolutionary effects on territorial defenders and sneaky copulators will keep occurring.

Territorial defense also extends to the level of the colony. Previous work suggests that nonhuman animals use signals (odors or sounds) to prevent conspecific noncolony members from entering a protected colony in both Hymenoptera and mole rate populations (Barker et al., 2021; Queller and Strassmann, 2002). If the signal detection system prevents unrelated individuals from gaining access to the colony, the defense is a barrier; if it reduces but does not prevent access, it is a restraint.

## 6 HUMAN POPULATIONS

Behavioral patterns in human cultures can be exploited for Darwinian purposes (Foley and Mirazón Lahr, 2011). We propose, in line with Dawkins and Krebs’s (1978) discussion of the relationship between “mind‐readers” and “manipulators,” that individuals in human societies will continuously develop strategies for both creating and avoiding barriers and restraints to exploitation. The forms these strategies take will depend both on population size and on receiver psychology, which develops because of both intrinsic and culturally learned biases (Soler et al., 2014). In human societies, where cultural information—maladaptive or otherwise—may travel quickly (Enquist et al., 2002), disequilibrium between competitors is likely to be common (Soler et al., 2014).

The full extent of human‐imposed barriers and restraints to exploitation is obviously too large to discuss here, but we believe that they range across cultures and relationships and that language in particular provides strategic individuals with virtually unlimited mechanisms for exploiting their own societies (Cronk, 1994). Some strategies that do not rely on language, however, may be universal among humans. For example, in a parallel way to begging in bird species, Lummaa et al. (1998) suggested that human infants may cry more vigorously to signal hardiness (Furlow, 1997) or to exploit parental caregiving. Because there is frequently a conflict between the desired level of care of offspring and the optimal amount of care required from a parent for ensuring the offspring's reproductive success, vigorous crying may be an exploitative measure for manipulating parents, or to outcompete potential siblings.

Within particular cultures, the mechanisms for thwarting exploitation will determine the most likely strategies individuals will develop to exploit others and their societies—a fact that may help to predict cultural exploitation before it takes place. The classic example of exploited reciprocity from Mauss (1925) shows that gift‐giving and reciprocity among the Maori is used for personal or familial gain: Individuals are known to provide gifts they know cannot be reciprocated. Assuming the custom was introduced as a mechanism to prevent free riding among Maori families, the strategy of over‐generosity to actively prevent reciprocity, and thereby damage a family's reputation, is a novel behavior that avoids the custom that may have previously functioned as a barrier to exploitation.

Numerous empirical studies and models reveal mechanisms for preventing exploitation associated with gift‐giving practices in hunter‐gatherer societies. Cronk (1989) distinguishes several hunter‐gatherer practices from the ostensibly “no‐strings attached” gift‐giving relationships seen in Western cultures. One practice that may be unusually effective at blocking exploitation is the “osotua” of Maasai pastoralists (Jacobs, 1965; Spencer, 1965); modeled by Aktipis et al. (2011). Osotua (translating literally to “umbilical cord”) relationships are all need‐based: individuals in need ask for help from those with resources, and those with resources give only so much as is needed. Models suggest that the osotua practice leads to longer group viability than does individual‐ or nonneed‐based pastoral systems. Cronk (2007) interviewed Maasai pastoralists about the behaviors that may lead to ending an osotua relationship, which included lying about needs, or lying about the resources one has to give. Interviewees added, however, that cheating in osotua relationships was “unthinkable.”

Numerous other examples, both in ethnographic data, laboratory experiments, and analytic and agent‐based models, suggest that social policing in humans, whether in the form of altruistic punishment and strong reciprocity (Fehr & Fischbacher, 2003; Gintis, 2000; also see Wiessner, 2005 for a case study in the Ju/’hoansi bushmen) through conditional cooperation via tag‐based signaling (Bruner, 2021; Riolo et al., 2001), or the evolution of social norms (Fehr & Schurtenberger, 2018), may have been important for maintaining social cohesion throughout our evolutionary history (Barclay, 2004; Barclay, 2013; Bliege Bird & Power, 2015; Gintis et al., 2001; Panchanathan & Boyd, 2004). These findings have led to support for the cultural group selection hypothesis (see Henrich, 2004; Henrich & Henrich, 2007; Henrich & Muthukrishna, 2021), which emphasizes the importance of group cohesion, altruistic punishment, and social norms for survival in between‐group competition and warfare, though Alexander (1985) develops a similar account without assuming intragroup cohesion. While these positions undoubtedly give proximate accounts for how barriers and restraints against exploitation—in the form of social norms, policing, and shared interest in group survival—prevent within‐group subversion, the possibility of novel strategies for exploiting one's own group by compromising such barriers and restraints needs to be assessed.

Across human populations and cultures, we believe that the possibility of novel strategies suggests that any barrier may become a restraint—or may fail to protect against exploitation altogether. The ingenuity of exploitative methods humans develop suggests that mechanisms for promoting group cohesion are not universally effective, even among successful groups. This is seen across societies, from hunter‐gatherer groups to urban cities.^1^ It may be, however, that social norms and socially transmitted beliefs about norms, as alluded to by the interviewee in the osotua system, can function as a barrier: Those who believe it is wrong to exploit others will not do so (for a discussion, see Gordon & Frank, 1990). Studies of human religions as signaling systems (see Irons, 2001; Sosis, 2003; Sosis & Alcorta, 2003) suggest, moreover, that communal belief and ritual helps to maintain cohesion and cooperation in human societies. In large societies, however, elites may exploit religious systems to control fellow adherents (Johnson, 2005), leading to an increase in religious skepticism (Cronk, 1994; Soler et al., 2014). Soler et al. (2014) argue that such skepticism will, in turn, lead exploiters of these systems to intensify manipulation of receiver psychology through a variety of signals. It may therefore be the case that larger, stratified societies are at an overall greater risk of being in disequilibrium, while in smaller societies, or smaller facets of larger societies, arms races may lead to barriers that put a cultural version of the Red Queen on hold, at least temporarily.

Features of language make clear, furthermore, the nuanced ways that the coevolutionary interaction between exploiters and honest signalers can drive complexity. For example, ubiquity of dialects and accents (Cohen, 2012), and the direct link between these features and individual preferences toward others (Anisfeld et al., 1962; Moffett, 2013), even from a very young age (Kinzler et al., 2007, 2009), suggests that vocal qualities are used for assortative cooperation (Cohen, 2012; Cohen and Haun, 2013; McElreath et al., 2003). Mimicry of these features may, furthermore, have selected both for a stronger ability to detect mimicry and for the use of signals that are more difficult to fake, both of which present a restraint against exploitation (Cohen, 2012). To the extent that mimics succeed, the defenses against mimicry will be restraints rather than barriers to exploitation.

Given that this potential mechanism for exploiting group preferences does not rely on the use of words with meaning, it is likely that strategies individuals may use complex language to evade or attenuate barriers and restraints to exploitation across cultures. This accords with the view that tactical deception or Machiavellian intelligence has been an essential factor in the evolution of primate intelligence (Byrne & Whiten, 1988; Humphrey, 1976). As mechanisms for deception and exploitation are ubiquitous, including in modern societies (Boyd & Mathew, 2015; Henrich, 2020; McNally et al., 2012), we suggest, following the notion that complex cognition permits more sophisticated strategies of exploitation, that applying barrier theory to human cultures helps to predict and expose such strategies as novel barriers for prevention are implemented.

## 7 PUTTING THE RED QUEEN ON HOLD

We suggest that the above review shows how barrier theory can apply to different types of exploitative systems; barriers can cause the Red Queen to rest, but novel strategies can overcome barriers and cause her to start moving again. The mechanisms for preventing exploitation and the ways a barrier can be overcome are determined by the system in question. In each of these examples, the relatively rapid evolution of germs and cancers permits the invading organism or growth to evade natural barriers. In the case of many cancers, tumor suppressor genes and cell adhesion may prevent oncogenesis unless countered by mutations or pathogens; in the case of infectious disease, germ evolution can lead to properties that allow an infectious organism to overcome the host's immune system; among animal populations, tactics, such as mimicry, are employed to overcome mechanisms that otherwise block exploitation. But selection, whether natural or cultural, determines whether and how barriers form—and how they are overcome.

In each of these examples, some exploiters are able to use methods for overcoming characteristics that are barriers to other exploiters. A commonality is heterogeneity among exploiters. Tumors, for example, rely on heterogeneity to proliferate: Once formed, the immune system and artificial treatments are not always effective for curing cancer, as the assaults on the disease select for cells naturally resistant to a particular therapy. High heterogeneity virtually guarantees the successful exploitation of the body, as even a single cancer clone with natural resistance may divide uncontrollably (Dey et al., 2017).

If it is possible that unknown strategies can be developed and used when rules are not strictly limited—unlike those seen in models of signaling games—then it is possible that, in any particular real‐world competitive relationship, one party will be exploited in an unforeseeable way, disrupting the potential for evolutionary stability. As barriers are generated in response to past exploitation strategies, exploiters who develop novel strategies for overcoming barriers to exploitation will have a fitness advantage; this may explain the evolution of the ability to overcome barriers by novel means, which can affect the trajectory of Red Queen‐like relationships: Unexpected strategies may allow one side to exploit the other, putting on hold, or perhaps ending entirely the coevolutionary relationship. In this way, barrier theory reveals how and why coevolutionary struggle develops into evolutionary stability, and vice versa.

Barrier theory emphasizes that the Red Queen stops running when an effective barrier evolves and further that this principle holds across populations and environments where exploitative relationships exist. Coevolutionary struggle resumes if the exploiter breaks through the barrier to transform it into a restraint. The Red Queen may never fully stop if the barrier‐breaking variant is present in at least one individual in the population of adversaries. If it is absent and each adversary is entirely dependent on the exploitation, the Red Queen stops running. This elimination of the exploitation, however, may be temporary if the adversary has others to exploit (e.g., other species), in which case there is a reservoir from which new exploiters may return with new characteristics that release them from being blocked by the barrier, causing the Red Queen to start running again. As discussed above for plant secondary compounds, however, the fact that the Red Queen may be perpetually on the run with some adversaries does not negate the fact that she may be able to stop running permanently against most of the potential adversaries, when they are blocked by a generally effective barrier.

We suggest, finally, that viewing coevolutionary struggles from the systems‐view of barrier theory reveals how innovative strategies may evolve to evade barriers and overcome restraints. This is because barriers and restraints reveal what strategies cannot be used, guiding the observer about what novel strategies may be developed (Table 1). Mimicry of honest signals and deception are, we hypothesize, common strategies for overcoming barriers in both natural and social systems. Future work, both modeling and empirical, should show the extent to which the principles of barrier theory hold across ecological and cultural environments and predict what strategies are likely to be developed to overcome mechanisms that thwart systemic exploitation.

**TABLE 1:** Summary of barriers and restraints that evolve through natural selection

Taxon/Taxa	Exploiter	Relationship	Barrier(s)	Restraint(s)
Multicellular organisms	Dysregulated; oncogenic parasites	Cancer; parasitism	Cell cycle arrest; cell suicide; cell adhesion; p53 protein	Slow division of dividing cells; restricted blood flow
Humans	*Plasmodium* agents of malaria	Parasitism	Duffy antigen receptor mutations; vaccination	Sickle cell anemia
Pine trees	Pine beetles	Herbivory	Secondary compounds that completely deter herbivores	Defensive chemicals (can be exploited by competitors)
Birds	Dishonest signaler chicks	Competition for parental care	Universal recognition of dishonest signals leading to dishonest signalers being ignored	Inconsistent recognition of dishonest signals
Cichlid and centrarchid fish	Males mimicking females, or signaling elevated social status	Competition for breeding resources	Complete deterrence of deceptive signalers by aggression	Partial success at deterring deceptive signalers
Colonies of Hymenoptera and mole rats	Access to colony by genetically unrelated individuals	Competition for colony resources	Elimination of unrelated individuals by aggression	Partial deterrence of unrelated individuals by aggression

## CONFLICT OF INTEREST

The authors have no competing interests to declare.

## Data Availability

Data sharing is not applicable to this article as no new data were created or analyzed in this study.

## ENDNOTE

^1^
Modern crime studies have started to make use of evolutionary reasoning in prevention work. Ekblom (1999), for example, has used the notion of coevolutionary struggle to emphasize changes in criminal tactics in the United Kingdom. He notes that, as new mechanisms to prevent crime are introduced, novel criminal behaviors inevitably develop; evolutionary insights from barrier theory can, therefore, help those involved in crime prevention to predict novel criminal tactics. Islam et al. (2019) extend this idea to security issues in technology.
